# Mr. Cuming, on Sphacelus

**Published:** 1804-04-01

**Authors:** Ralph Cuming

**Affiliations:** Romsey


### *To the Editors of the Medical and Physical Journal.*

GENTLEMEN,

IT must ever be the wish of the enlightened and liberal part of the profession, to impart for the good of mankind, that knowledge, which their practice and experience teach them to be worthy the attention of the medical world; and in my opinion the present critical juncture of affairs in a peculiar manner proclaims the necessity of contributing one's mite towards that fund which constitutes the methodus medendi in the department of Surgery.

### *On Sphacelus.*

Two cases of this kind have lately come within my notice, wherein soda nitrata or nitre, has completely checked the progress of mortification. The first time I had recourse to this remedy, (which may justly be termed a sovereign one) was whilst I was Surgeon of His Majesty's Ship St. George; it was applied to a man's foot which was wounded with a cannon shot in an action near Cadiz; a mortification took place soon after the accident, owing to the irritation of splinters or spiculæ impacted in the tendinous and nervous parts, (so small as not to be discovered at first) and the destruction of the soft parts. The usual remedies in such cases were had recourse to; such as washing the mortified parts with spt. terebin. vini, &c. vinegar, and fomentations of bark, but without perceiving any benefit resulting from their application. The fœtor emanating from the wound, which was black and cadaverous, was not at all corrected, nor was any separation of the sphacelated parts perceptible; on the contrary, the disease was spreading rapidly all over the foot. In this dilemma revolving in my mind the various antiseptics which the materia medica afforded, I luckily thought of nitre, and began to use it very very finely pulverized in large quantities, sprinkling it all over the wound, covering it so perfectly, as to enable me to make some degree of pressure without soiling my fingers, for the purpose of promoting its union with the mortified parts; the wound was dressed three times a day as the climate was warm, and the danger from absorption consequently great. In the course of twelve hours the disagreeable stench issuing from the wound, which before was so extremely offensive as to make the nurses sick, was now quite gone, the extent of the mortification was soon discovered by a line of separation surrounding the wound; here the beneficial effects of the nitre were very evident both as far as related to its antiseptic as well as stimulant power, the former in subduing putrefaction, the latter by restoring energy and activity to the living parts, sufficient to enable them to throw off the causa nocens.

It may seem unnecessary to mention that as much of the sphacelated parts as could be removed by the assistance of the scissars and forceps, were taken away at every dressing, and the wound well washed, sometimes with vinegar, at other times with decoction of cinchona. By perseverance and strict attention to the above remedies, particularly the nitre, though the patient was reduced to the lowest ebb of human misery, which was sufficiently evinced by symptoms the most distressing and alarming, such as yellowness of the skin, a black furred tongue, tetanus, and some degree of locked jaw, extreme debility, quick and fluttering pulse, he was restored to health.

I found in this case, as I have done in many others, the exhibition of bark in substance do great mischief, though not given to near the amount recommended by some medical writers. One would imagine some medical authors were afraid of deviating from the paths of their predecessors, when they are found slavishly subscribing to their rules, whilst the conviction of their own experience should teach them the folly, and even criminality, of recommending medicines, which cannot boast of that consequence or efficacy, which they have laboured to bestow upon them.

It generally happens, as it did in this instance, that, though the patient previously to its administration, shall have some appetite, it soon fails, and the space of twelve hours entirely deprives him of all inclination to eat; his fever increases, and he loaths every thing offered him in the shape of food. This is not the worst consequence, the medicine at length proves so nauseating and disgusting, that the stomach, as if conscious of the violence done to it, arms itself with the resolution of rejecting this inhospit-
able able and noxious tenant. Such is the remedy employed by the vis medicatrix naturæ, to rid herself of matter so heterogeneous and offensive to this delicate organ, the primum mobile of the system. Nor can it be wondered at that this should happen, when you reflect on the indigestible nature of bark, however finely it may be pulverized; the human stomach is not like that of the ostrich, it will not digest iron; and it does not require much penetration to discover, that the digestive faculties of this noble organ are greatly impaired by disease: then how can it be supposed that a substance, which may with the greatest propriety be termed foreign, should not prove injurious, when other things, mild and congenial to it in a healthy state, are now offensive?

It is not my wish to have wrong inferences deduced from these observations, I have said enough to convince that I am inimical to the exhibition of bark in substance; yet I would not have it imagined that it cannot be employed with advantage in any form; indeed, I think far otherwise, for I am persuaded it is of infinite service when given in decoction, when you can augment or diminish its strength ad libitum, without clogging up the stomach with a mass of heterogeneous, indigestible matter; an advantage which will appear to every unprejudiced mind, of the greatest importance, as far as relates to the welfare of the patient and success of the surgeon, whose anxiety must ever be commensurate with the danger and difficulty of the case.

The other instance wherein the same plan of cure was adopted with success, was a mortification of the leg, originating from an ulcer, which degenerated into this state, from poor living and bad treatment of a pauper belonging to this place.

I am, &c.

*Romsey, March* 14, 1804. RALPH CUMING.

